# High-resolution dynamic full-field optical coherence microscopy: illuminating intracellular activity in deep tissue

**DOI:** 10.1038/s44303-026-00153-y

**Published:** 2026-03-31

**Authors:** Erikas Tarvydas, Austėja Trečiokaitė, Egidijus Auksorius

**Affiliations:** Center for Physical Sciences and Technology (FTMC), Vilnius, Lithuania

**Keywords:** Biological techniques, Engineering, Optics and photonics

## Abstract

Dynamic full-field optical coherence microscopy (*d*-FF-OCM) is a label-free imaging technique that captures intrinsic subcellular motions to generate functional contrast. This dynamic approach yields images with fluorescence-like contrast, highlighting active structures without the need for fluorescent labels. However, current *d*-FF-OCM implementations have limitations when imaging deep within highly scattering tissues at high resolution. Here, we present a new high-resolution *d*-FF-OCM system that overcomes these limitations, enabling depth-extended high-resolution imaging in such tissues. The setup uses 100× oil-immersion objectives (NA = 1.25) and a high-brightness, laser-pumped incoherent white light source to achieve nanometre-scale resolution at depths up to ~120 µm in highly scattering samples. We also incorporate real-time reference arm adjustment to maintain signal strength and contrast as the focus moves deeper into the sample. Using this system, we imaged fresh ex vivo mouse liver and small intestine with extended depth and detail. In these tissues, the dynamic contrast clearly revealed fine structures not visible with conventional OCT—for example, the sinusoidal microvasculature and organized cell layers in the liver, as well as neural plexuses and crypts in the intestine—all visualized label-free.

## Introduction

Achieving high-resolution, label-free imaging deep within highly scattering biological tissues remains a major challenge in optical microscopy^1,2^, critical for understanding fundamental biological processes and advancing clinical diagnostics. Optical Coherence Tomography (OCT) is a powerful imaging technique that has continuously evolved to achieve higher resolution and imaging depth^3^. While conventional scanning OCT modalities, including micro-OCT (*µ*OCT), which achieves ~1–2 µm resolution^4^, offer deep tissue penetration, they inherently face a trade-off between lateral resolution and depth of focus, limiting their utility for high-speed and high-resolution volumetric acquisition^5^. Full-Field Optical Coherence Microscopy (FF-OCM) decouples lateral resolution from depth of focus by axial (*z*) scanning and camera-based parallel detection of *xy* planes enabling much higher resolution^6–10^, albeit at the expense of slower volumetric acquisition due to the need for axial scanning. Despite the slow imaging speed of FF-OCM, for example, in vivo imaging of the eye has been successfully demonstrated^11^. Fourier-domain FF-OCM advances imaging speed^12^, and in some cases surpasses that of scanning OCT, but it remains constrained by the same resolution-depth trade-off and exhibits reduced sensitivity. Nevertheless, its ability to computationally correct for optical aberrations enables superior resolution for in vivo retinal imaging compared to standard scanning OCT^12,13^. Recently, pupil-scanning in Fourier-domain FF-OCM enabled 3D imaging on an ex vivo opaque cornea at a resolution of 290 nm and 500 nm in the lateral and axial planes, respectively^14^. However, such approaches remain to be validated in highly scattering tissues (e.g. liver), and their ~1 s per volume rate, while remarkable for volumetric imaging, may limit its suitability for applications where rapid 2D image acquisition is required, such as dynamic OCT. Similarly, other emerging techniques, like variants of quantitative phase imaging techniques^15^ have demonstrated nanoscale topographic mapping and refractive index reconstruction in cornea. However, these methods primarily focus on structural and optical property quantification rather than intrinsic cellular dynamics, and their application to highly scattering tissues like liver, particularly for rapid dynamic imaging, remains to be fully explored. Beyond its spatial resolution, OCT images suffer from contrast that is often nonspecific, making it difficult to distinguish different tissue components. To overcome this limitation, a variety of functional contrast techniques—such as OCT angiography^16^, elastography^17^ and dynamic OCT^18^—have been developed. The latter is the simplest of them, as it does not require additional elements, but provides a great contrast improvement in live tissue by using temporal fluctuations in the OCT signal caused by intrinsic subcellular dynamics, such as metabolic processes, cytoskeletal movements, and organelle activity^18^. These fluctuations generate a fluorescence-like contrast, suppressing signals from static, highly scattering structures and highlighting active cellular components^19^. Although these vibrations can be rapid, their frequency remains stable for hours, making them a reliable contrast mechanism, and thus, a marker. It has been elegantly demonstrated in dynamic Full-Field Optical Coherence Microscopy (*d*-FF-OCM)^20^ by simply switching the piezo performing the phase-shifting *off* and relying on the subcellular tissue motion to generate the signal. This dynamic contrast enables *d*-FF-OCM to resolve cellular morphology, distinguish cell types, and visualize processes at high resolution, such as mitosis, rosette formation, cell motility, and intracellular dynamics^7,8,20^. Beyond biological research, *d*-FF-OCM has shown promise in biomedical applications, including rapid intraoperative cancer diagnostics^21–24^. Despite these capabilities, *d*-FF-OCM, like all optical microscopy techniques, can benefit from improvements in spatial resolution and imaging depth to enable the observation of smaller subcellular structures deep in tissue. To this end, some studies have employed NA ≈ 0.8 lenses in *d*-FF-OCM^6,7,25^. However, advancing to even higher numerical apertures (e.g., NA = 1.05 for transparent retinal explants^25^) presents challenges, primarily dispersion mismatch and insufficient light intensity at the sample, both of which reduce SNR. When two high-NA, highly corrected objective lenses are employed in a Linnik interferometer, particularly with a broadband light source like white light, chromatic dispersion mismatch between the objectives can reduce the contrast of the interference fringes, which in turn reduces OCT signal and thus SNR. Furthermore, illuminating a large field of view (FOV) through high-NA objectives with LEDs, which are used for its large spatial and temporal incoherence, is challenging. LEDs have high *étendue* and low radiance, making it difficult to efficiently fill the objective’s pupil and deliver sufficient light intensity across the FOV. Therefore, previous *d*-FF-OCM systems have relied either on LEDs, which are spatially incoherent but too dim to deliver sufficient irradiance through a high-NA objective, or—more rarely—on laser-based sources, which are bright but introduce speckle due to their high spatial coherence^26^. The highest reported intensity on a sample in *d*-FF-OCM using LED was 31.8 W/cm^2^ (with 810 nm central wavelength and 25 nm bandwidth)^8^. This level was too low to operate a high-full-well capacity camera near saturation level at the adequate reference strength. Although high–spatial-coherence lasers perform well in scanning OCT^4,27^, in FF-OCM they produce strong speckle artifacts that degrade image quality—especially at greater depths—thereby limiting both penetration depth and SNR. In Fourier-Domain FF-OCM^28^ and even in scanning OCT^29^ coherent noise can be reduced by partially decreasing spatial coherence or employing other means^30^. However, in FF-OCM, spatial coherence should be reduced as much as possible to maximize coherence-noise suppression, while simultaneously and uniformly filling the objective’s pupil and covering the full field of view.

In this study, we employ 100× oil-immersion objectives (NA = 1.25) and a high-brightness, laser-pumped incoherent white light source to achieve ~270 nm lateral and 500 nm axial resolution, and imaging depth of ~120 µm in highly scattering samples. By delivering an irradiance of up to 170 W/cm²—5.3 times higher than achieved previously for lower-NA systems^8^—this source provides the necessary signal strength to resolve subcellular dynamics deep within tissue. This enables depth-extended high-resolution imaging, free from coherent artifacts. Imaging depth is further extended via real-time reference arm adjustment^31^. We illustrate its superior performance on ex vivo mouse tissue, such as liver and intestine.

## Results

### Resolution

We have evaluated lateral and axial resolutions to be ~270 nm and ~500 nm, respectively, using USAF resolution target (HIGHRES-2, Newport), as shown in Supplementary Fig. S1a, b. We have also obtained a similar result for lateral resolution by analysing *d*-FF-OCM image of a liver with Fourier Ring Correlation (FRC) method^32^, as shown in Supplementary Fig. S1c, d. At the maximum reported imaging depth of 120 µm the resolution decreases to about 1 µm, as evaluated by the FRC method and shown in Supplementary Fig. S1f.

### Liver imaging

**Fig. 1:**
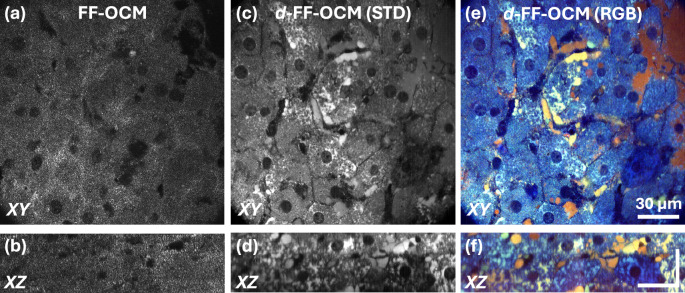
Dynamic FF-OCM shows superior contrast over static FF-OCM. High-resolution *en face* (*XY*) and axial (*XZ*) views of fresh mouse liver acquired with conventional FF-OCM (**a**, **b**) versus dynamic FF-OCM (**c**–**f**). Dynamic imaging (**c**–**f**) enhances visualization of hepatocyte plates and cell-filled sinusoids. In the dynamic panels (**e**, **f**), blue denotes low-frequency (1–2 Hz), green mid-range (3–100 Hz), and red high-frequency (100–250 Hz) activity. Scale bar: 30 µm.

The fresh liver sample was cut in half and put on a coverslip with its cut side facing the coverslip. We then imaged it using both static and dynamic acquisition modes and two contrast-generation algorithms (STD and FFT) to extract the signal. Figure 1a shows a static (conventional) *en face* FF-OCM image acquired in ~1 s that matches the acquisition time used for dynamic FF-OCM acquisition of 512 raw images for direct comparison. *En face* (Fig. 1a) and axial (Fig. 1b) images reveal the dense packing of hepatocytes (20–30 µm diameter) with barely visible boundaries in between and offer little subcellular detail beyond dark nuclear voids. By contrast, *d*-FF-OCM processed via the temporal STD method (Fig. 1c, d) produces a boost in contrast: cell borders and intracellular structures become clearly visible; filamentous networks of low-frequency fluctuations—likely related to mitochondrial dynamics^20^—emerge throughout many hepatocytes, alongside nuclear activity. Applying an FFT-based spectral segmentation to the same time series yields RGB-coded images (Fig. 1e, f) that further differentiate tissue components: hepatocyte bodies map predominantly to low-range frequencies, whereas sinusoids—capillary-like vessels found in the liver – map to a broader and higher range of frequencies.

**Fig. 2:**
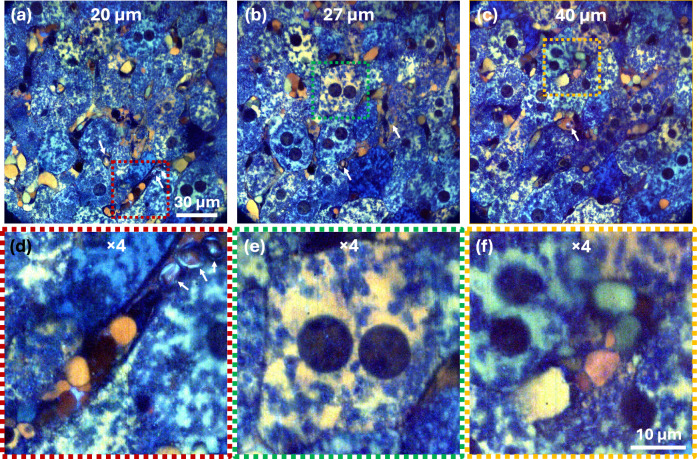
Imaging subcellular dynamics across depth in fresh ex vivo mouse liver with dynamic FF-OCM. Images taken from Supplementary Movie S2 correspond to the images recorded at depths of 20 µm (**a**), 27 µm (**b**), and 40 µm (**c**), clearly show hepatocytes and sinusoids throughout the tissue. Panel (**d**) is a 4× magnified view of the red box in (**a**), highlighting a blood-filled sinusoid with visible erythrocytes (white arrows) and other cells. Panel (**e**) is a 4× magnified view of the green box in (**b**), showing a binucleated hepatocyte whose cytoplasm exhibits heterogeneous dynamic activity and whose nuclei also display motion. Panel (**f**) is a 4× magnified view of the yellow box in (**c**), featuring a sinusoid containing putative small blood elements—likely platelets and immune cells. In all the panels, colour encodes subcellular dynamics: blue (1–2 Hz), green (3–100 Hz), and red (100–250 Hz).

Sinusoids exchange substances between blood and hepatocytes^33^ and are lined by endothelium and populated by erythrocytes, immune cells and other substances, like triglyceride-rich lipoproteins (chylomicrons) and plasma. Dynamic FF-OCM images in RGB of deeper tissue layers (20–40 µm below the surface) of the same specimen are shown in Fig. 2a–c. It shows continuous cords of hepatocytes interlaced with sinusoids throughout this depth range (See Supplementary Movies S1, S2). Four-times-magnified insets (Fig. 2d, f) highlight blood-filled sinusoids with discernible erythrocytes (Fig. 2d), a binucleated hepatocyte exhibiting spatially heterogeneous cytoplasmic and nuclear dynamics (Fig. 2e), and sinusoids containing different structures within. These structures, for instance, could be platelets and immune cells such as Kupffer, NK or T cells, based on size/morphology. The two smallest structures in the sinusoid, seen in Fig. 2f, appearing in light and dark red, are 1.65 × 3.3 µm and 2.5 × 4.2 µm in size, respectively. Importantly, high-contrast dynamic imaging persists even down to 120 µm, as demonstrated in the axial (XZ) views of Supplementary Fig. S2e, f; for example, the 80 µm slice in Supplementary Fig. S2d still resolves hepatocyte and sinusoidal activity across a broad frequency spectrum.

**Fig. 3:**
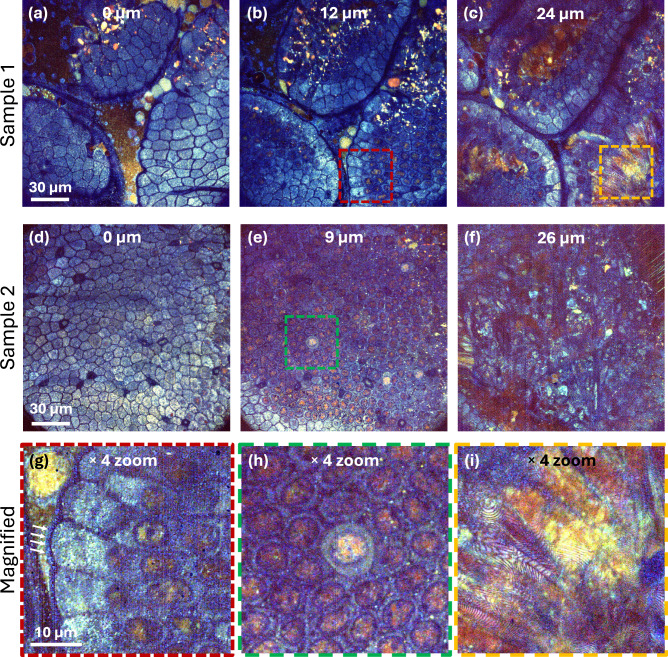
Dynamic FF-OCM reveals depth-dependent subcellular architecture and activity in ex vivo mouse intestinal mucosa. *En face* images at three increasing depths are shown for sample #1 (**a**–**c**) and sample #2 (**d**–**f**), taken from Supplementary Movies S3, S4, illustrating the epithelial layer overlying the lamina propria. Panels **g**–**i** present 4× magnified detail views of key features from these depth series: **g** epithelial microvilli (white arrows) and nuclei; **h** a putative goblet cell among enterocytes; **i** mesenchymal elements within the lamina propria. Dynamic fluctuations are colour-coded by frequency: blue (1–2 Hz), green (3–100 Hz), red (100–250 Hz).

**Fig. 4:**
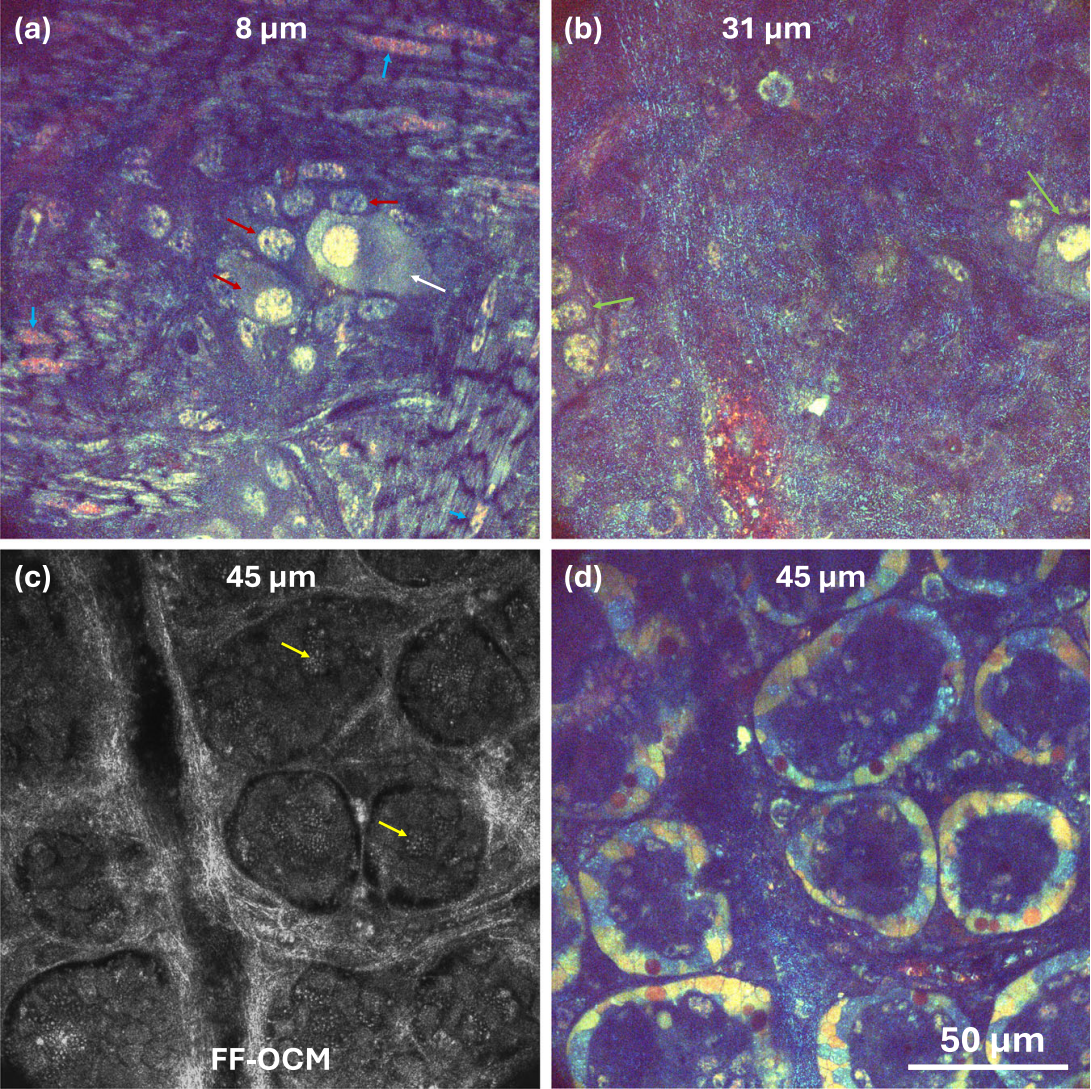
FF-OCM of mouse gut from the serosal side reveals enteric plexuses and crypt architecture (taken from Supplementary Movie S5). **a** *d*-FF-OCM of the myenteric plexus showing neurons (white arrow), glial cells (red arrows), and adjacent longitudinal and circular muscle fibres with visible nuclei (blue arrows). **b** *d*-FF-OCM of the submucosal plexus highlighting neuronal and glial cell bodies at the image edges (green arrows). **c** Static FF-OCM of intestinal crypts visualizing Paneth cells and their characteristic granules (yellow arrows). **d** *d*-FF-OCM of the same crypt region revealing epithelial cells and underlying submucosal blood vessels. Dynamic fluctuations are colour-coded by frequency: blue (~1–2 Hz), green (2–50 Hz), red (50–250 Hz).

To demonstrate the versatility of this method, we imaged the mouse small intestine from both the mucosal and serosal sides (Figs. 3 and 4, respectively).

### Small intestine imaging (mucosal side)

Figure 3 presents images of two mouse gut samples imaged from the mucosal side, highlighting the villi (see Supplementary Movies S3 and S4 for full depth imaging). Epithelial cells, predominantly enterocytes, are organized in a mosaic pattern at the villus apex, as depicted in Fig. 3a, d. Nuclei of epithelial cells are clearly visible in Fig. 3b, e and in magnified view in Fig. 3g that also show structures consistent with microvilli on the epithelial cells. In addition to enterocytes, other cell types—putative goblet cells—are visible in Fig. 3e and in the magnified view in Fig. 3h. Highly active cells within the villi, located in the lamina propria, are shown in Fig. 3c and in magnified view in Fig. 3i. These cells are likely antigen-presenting cells, lymphocytes, and mesenchymal elements of the intestinal lamina propria, such as myofibroblasts, fibroblasts, and mural cells^34^. Finally, features suggestive of vascular networks are visible in Fig. 3f.

### Small intestine imaging (serosal side)

Figure 4 presents images of the mouse small intestine acquired from the serosal side. Figure 4a depicts the myenteric plexus^35^ (neurons and glia) located between the longitudinal and circular muscle layers (Supplementary Movie S5). This localization has been demonstrated previously with static and dynamic FF-OCM^36,37^. The two muscle layers can also be identified in the Fig. 4a oriented perpendicular to each other. Figure 4b shows cells at 31 µm, embedded in the connective tissue of the submucosa, that also look like neurons and glial. Given that the myenteric plexus already appears at 8 µm depth, the plexus shown here is most likely submucosal, particularly because we observe large blood vessels within it (Fig. 4b–d; Supplementary Movie S5), which are characteristic of the submucosal plexus^35^. This dataset is unique in capturing both networks in the same *z*-stack. Figure 4c highlights Paneth cells, specialized epithelial cells of the small intestine^38^, located at the base of intestinal crypts.

These cells are identifiable in standard FF-OCM images (Fig. 4c) by their readily discernible secretory granules, characteristic of Paneth cells, which are the only granule-containing cell type at the crypt base. The Paneth cells and their granules were even more visible in fixed mouse gut, as shown in Supplementary Fig. S3 and Supplementary Movie S6. Figure 4d shows *d*-FF-OCM image of round, ring-shaped profiles (the crypt lumens) surrounded by a band of brightly coloured cells—crypt epithelial cells. Spindle-shaped inter-crypt structures can also be seen in Supplementary Movie S5 that could be subepithelial myofibroblasts (and fibroblasts). Figure 4c, d provides complementary information - structural and metabolic insights into the crypts, respectively.

## Discussions

We achieved ~270 nm lateral and 500 nm axial resolution (Supplementary Fig. S1) using 100× oil-immersion objectives and a bright laser-pumped white light source. The high NA objective also enabled more efficient light collection from a sample, which together with the high source brightness allowed collecting more photons and increasing SNR. Furthermore, real-time reference arm adjustment ensured coherent and confocal gate overlap, producing signal (Supplementary Fig. S1e) and high contrast images (Supplementary Fig. S2) up to 120 μm in fresh ex vivo mouse tissues, corresponding to 5–6 hepatocyte layers. Using a conventional white LED results in a shallower imaging depth when compared to the light source used in this work, as shown in Supplementary Fig. S4. Moreover, the source enabled 500 fps acquisition instead of the typical 100 fps in *d*-FF-OCM. This faster imaging rate allowed frequency analysis extension from up to 30–50 Hz^6,20,24,25,39^ to up to 250 Hz. This extension allowed the capture of faster dynamic processes and generation of more contrasted *d*-FF-OCM images, as illustrated in Supplementary Fig. S5 and Supplementary Movie S7. For example, some of the structures with 0–30 Hz analysis in Supplementary Fig. S5e are not well separated but become separated with 0–250 Hz analysis, shown in Supplementary Fig. S5f. Also, as shown in Supplementary Fig. S5g, frequency spectrum extends well above the usual 30–50 Hz range for all structures. While RGB segmentation (for example, blue: low ~1–3 Hz; green: mid 3–100 Hz; red: high 100–250 Hz) offers intuitive visualization, it relies on arbitrary binning and may not fully capture spectral essence. However, we find that different ranges produce similar RGB images, as shown in Supplementary Fig. S6a–c and Supplementary Movie S8, suggesting that such image generation is relatively stable. Alternatives like HSV mapping, autocorrelation decay, or singular value decomposition (SVD)^40^ could provide more comprehensive representations, though potentially at the expense of interpretability. These improvements in resolution and vibration spectrum analysis allowed us to better resolve filamentous networks in hepatocytes (likely related to mitochondrial dynamics^20^) and heterogeneous nuclear activity (Figs. 1 and 2) in liver. Sinusoids displayed broadband, high-frequency signals, enabling visualization of erythrocytes and putative immune cells (e.g., Kupffer or NK cells, based on size, morphology, and location) at depths up to 120 μm (Supplementary Fig. S2), which is in contrast to previous *d*-FF-OCM^20,41^ and scanning dynamic OCT^42–46^ studies on liver that do not clearly visualize sinusoids or distinctly resolve individual hepatocyte layers, likely due to limited SNR or spatial resolution. In small intestine, we captured microvilli, putative goblet cells, and lamina propria elements from the mucosal side (Fig. 3), which is comparable to two-photon autofluorescence imaging of metabolic activity through detection of NADH and FAD signal^47,48^. Compared to *d*-FF-OCM on human mucosa^49^, our results show superior subcellular detail. From the serosal side, we report the first OCT images of Paneth cells (Fig. 4c) that are especially clearly visible when tissue is fixed (Supplementary Fig. S3, Supplementary Movie S6). We could also identify the submucosal plexus (Fig. 4b, Supplementary Movie S5). These results complement prior lower-resolution views of the myenteric plexus using FF-OCM^36^ and *d*-FF-OCM^37^. A comparison between static and dynamic images acquired at the crypt base reveals cellular populations distinct from Paneth cells. Given their localization within the crypt, these additional cells may represent stem cells^50^. The epithelial cells within the crypts are clearly discernible (Fig. 4d). Future studies should aim to identify their specific types, which may exhibit heterogeneity, using correlative imaging, such as fluorescence imaging. Although dual FF-OCM and fluorescence microscopy systems can be readily developed^51^, the integration of *d*-FF-OCM with two-photon microscopy^52,53^ would be most valuable. This is due to two-photon microscopy’s superior depth penetration with intrinsic optical sectioning and reduced phototoxicity, providing a powerful complement to *d*-FF-OCM dynamic contrast. Contrast-to-Noise Ratio (CNR) analysis, shown in Supplementary Fig. S5h, i reveals that CNR does not saturate at 250 Hz, and thus, even higher frequencies could be beneficial to further improve dynamic RGB images. Further frequency expansion beyond 250 Hz could be achieved by utilizing a faster camera, as higher-frequency components of cellular dynamics may lie within the vibrations of structures such as nuclei and other organelles. For example, there is indications that there is significant degree of signal around 3 kHz^54^. While the system could be further improved with even higher NA objectives, such as, for example, NA = 1.45, and a brighter, spectrally broader light source, it is already operating near optimal parameters. Increasing NA would require more complex objective lenses, potentially complicating chromatic dispersion matching and raising costs significantly (from ~€1500 for current objective to >€10,000 for the highest NA one). Expanding spectrum to, for example, 400 nm would exacerbate the dispersion mismatch, and moreover, introduce more phototoxic light to samples. Similarly, additionally increasing intensity on a sample—via higher power or brightness of the source—would raise phototoxicity risk even without more harmful 400–500 nm spectral part. Although tissues like liver tolerate higher powers than isolated cell lines, thanks to extracellular matrix support, superior heat dissipation and the absence of labelling, further increases in power could prove counterproductive. Nonetheless, the use of 50 mW of illumination power over a 173 µm × 173 µm field of view—corresponding to an irradiance of 170 W/cm²—may be acceptable for imaging a variety of tissues. For comparison, continuous exposure to 561 nm light at 1000 W/cm² for several minutes has been used to excite fluorescence in live NIH 3T3 cells without any observed adverse effects^55^. Moreover, imaging mitochondria—one of the main contrast sources in dynamic OCT—has been performed at irradiances as high as 10 kW/cm² at 561 nm for up to one minute before noticeable morphological changes occurred^56^. No signs of phototoxicity or metabolic rate change were observed in the reported results (see Supplementary Fig. S2e), despite unnecessarily prolonged light exposure during image saving, which took five times longer than the 1-second acquisition. By using multiple NVMe SSDs in RAID 0, we can achieve up to ~4× higher I/O throughput, reducing saving to <1 s per 512-frame stack, which results in 3–4 times shorter acquisition of the entire *d*-FF-OCM stack. Turning off illumination during saving, as the light source supports triggering at rates of at least 100 kHz, could further minimise potential phototoxicity to the sample. Although the current results of live ex vivo tissue are primarily of interest for fundamental biological research, the imaging approach introduced here could also be adapted for in vivo applications. The combination of high spatial resolution, dynamic contrast, and extended imaging depth—achieved through bright, spatially incoherent illumination and high-NA objectives—provides a foundation for live tissue imaging. in vivo implementation would require overcoming challenges such as physiological motion and optical access, but these are addressable with established techniques like motion gating and water-immersion objectives. Similar challenges have been already tackled in *d*-FF-OCM systems with lower NA configurations^40^. Considering higher SNR and imaging rate achieved here, the method has the potential to enable high-contrast, label-free visualization of cellular dynamics in live animal models or clinical contexts.

In conclusion, the reported system uses high-NA optics, a bright incoherent source, and extended frequency analysis to enable improved *d*-FF-OCM imaging of depth-extended dynamics. By visualizing novel structures in mouse liver and intestine with fluorescence-like contrast, it holds promise for biomedical research and various clinical applications. Ultimately, *d*-FF-OCM is poised to play a role in advancing personalized medicine, enabling earlier and more precise diagnostics, and facilitating a deeper understanding of disease mechanisms in a dynamic, living context, particularly for applications like rapid intraoperative pathology where its label-free, high-resolution capabilities are invaluable.

## Methods

### Optical system

**Fig. 5:**
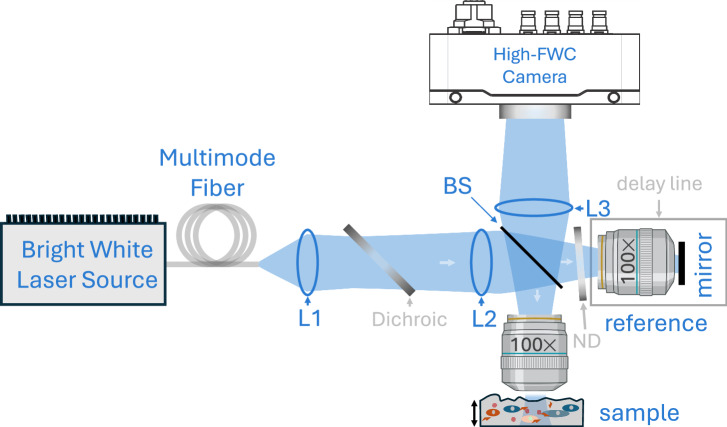
Schematic of the dynamic FF-OCM system featuring two 100× oil immersion objectives in the interferometer and a bright laser-driven incoherent white light source. The light is delivered to the microscope by a multimode fiber and sent to an interferometer composed of a 50/50 beamsplitter and the objectives. Interferograms are detected by a high full well capacity (FWC) camera.

The dynamic FF-OCM system, illustrated in Fig. 5, is comprised of three primary components: a high-brightness incoherent white light source (LS–WL1, Lightsource.tech, Germany), a Linnik interferometer with matched high-numerical-aperture objectives (Plan N 100×, NA = 1.25, Olympus, Japan), and a high full-well-capacity (FWC) camera (Q–2HFW–CXP, Adimec, Netherlands). The light source is a bright, high-power and compact unit that generates white light via the fluorescent conversion of focused 450 nm laser light on a specialized converter phosphor. A maximum of 300 mW was coupled into a multimode fibre (1 mm core, NA = 0.39) and delivered to the system, where after collimation with lens L1 (*f* = 25 mm), the light is spectrally filtered to remove the strong 450 nm laser peak using a dichroic beamsplitter (DMLP490R, Thorlabs) with a 490 nm cut-on wavelength. Lens L2 (*f* = 150 mm) forms the image of the fibre tip onto the pupil planes of both 100× objective lenses. The resulting 6× magnification of the fibre tip onto the pupil planes of the objective lenses ensures proper filling, which is crucial for achieving optimal resolution. The light is then distributed between the sample and reference arms by a 50/50 beamsplitter. Up to 50 mW could be sent onto the sample through the objective. The sample was mounted on a translation stage equipped with a stepper motor (Zaber T-NA08A25-S) for axial scanning. The entire reference arm was mounted on a separate motorized translation stage, enabling automatic optical path length adjustment. This adjustment keeps the temporal and confocal gates aligned at greater depths, counteracting the effect of the refractive-index mismatch between tissue and immersion oil^31^, and thus maintaining imaging up to 120 µm depth. A mirror between the objective lens and the beamsplitter – that sends the light down (not shown in Fig. 5) in the sample arm was mounted on a piezo stack (Piezomechanik STr-25) to enable controlled phase-shifting for deriving static (conventional) FF-OCM images. The reference arm was attenuated to 10% (in the double pass) by inserting an OD 0.5 neutral density (ND) filter to optimize the SNR. An additional ND filter of the same thickness, but with 0 OD, was inserted into the sample arm to compensate for chromatic dispersion. The beamsplitter then recombined light from the sample and reference arms, and a tube lens L3, *f* = 180 mm (SWTLU-C, Olympus) focused the resulting interferogram onto the camera. To obtain uniform *en face* sections, a small tilt was applied to the sample to compensate residual path-length mismatch and maximize interferometric contrast across the field of view.

### Image acquisition and processing

**Fig. 6:**
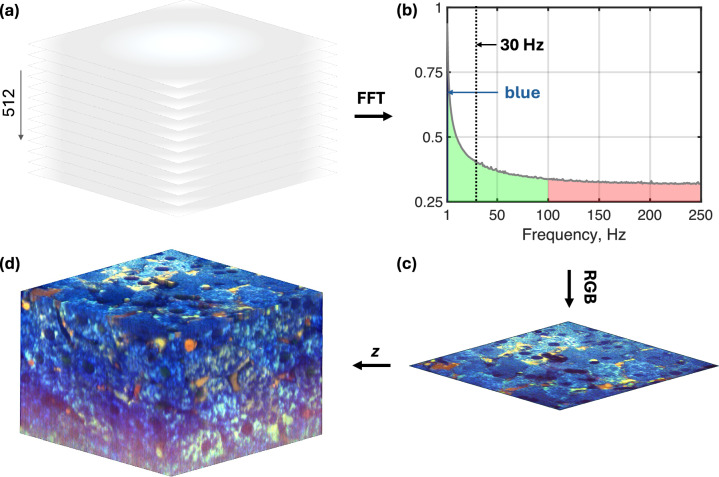
Generation of dynamic FF-OCM images. To generate dynamic image, a stack of 512 raw images (**a**) is fast Fourier transformed (FFT) pixel-by-pixel to generate frequency spectrum (**b**) for each pixel. The spectrum in (**b**) represents the average over all pixel spectra from the image in (**c**). The spectrum in (**b**) is segmented into low (blue: 1–2 Hz), mid (green: 2–100 Hz), and high (red: 100–250 Hz) frequency bands. The blue (1–2 Hz) band is barely visible—see the blue arrow. Vertical line shows the upper frequency cut (of 30 Hz) that were used in most of the previous studies; **c** presents an RGB image with each pixel coloured according to its relative spectral content; **d** shows one projection of 3D volumetric rendering (see Supplementary Movie S1 for full video) resulting from the RGB images generated at multiple *z* positions, spanning 100 µm.

The camera acquired 1440 × 1440 images at 500 fps, with each pixel optimized for 2 million electrons full well capacity detection. The imaging field-of-view (FOV) was 173 µm × 173 µm with pixel size of 120 nm in the sample plane. Data transfer from the camera to the computer was managed by a BitFlow Cyton-CXP4 PCIe CoaXPress frame grabber, capable of transferring data at up to 25 Gb/s. Instrument control and synchronization were implemented via a National Instruments NI-PCIe6363 DAQ card. Data acquisition and analysis was managed by a custom LabVIEW application. Static FF-OCM images were derived by calculating a square root of *(I*_*1*_ *– I*_*3*_*)*^*2*^ + *(I*_*2*_ *– I*_*4*_*)*^*2*^, where *I*_*1*_*, I*_*2*_*, I*_*3*_ and *I*_*4*_ are images acquired at *0°*, *90°*, *180°* and *270°* phase changes imparted by different positions of the piezo. Dynamic FF-OCM images were acquired by deactivating the piezo-driven phase-shifting and instead relying on intrinsic subcellular movements within the tissue. These movements induce temporal intensity fluctuations on the camera, occurring on a millisecond timescale. Figure 6 illustrates how the raw image sequence is processed to generate the dynamic images. A 512-frame stack is acquired (Fig. 6a), and each frame is normalized to correct for camera’s nonlinear response and light source fluctuations. For each pixel, its 512-point intensity time series is then analysed in two ways: (i) computing the temporal standard deviation (STD) and (ii) performing a Fast Fourier Transform (FFT). STD analysis yields a single value per pixel, generally reflecting fluctuation strength. FFT analysis, conversely, provides a frequency spectrum for each pixel (Fig. 6b). For visualization, we here arbitrarily integrate each pixel’s fluctuation spectrum into three bands—low (1–3 Hz), mid (3–100 Hz), and high (100–250 Hz)—which we map to blue, green, and red channels, respectively. These boundaries were chosen to emphasize slow, intermediate, and fast subcellular motions, but they can be adjusted to suit different samples or analysis goals. The integrated values were *log*-transformed and were assembled into a composite RGB image (Fig. 6c). Before the RGB image generation, to enhance overall contrast, the highest 1% of pixel values in R, G and B channels were saturated to full intensity, and, conversely, the lowest 1% of the values in those channels were set to zero intensity. For volumetric RGB rendering (Fig. 6d), we repeated this process over 100–250 *z*-steps, each taking ≈1 s to acquire and ≈5 s to save.

To correct the mismatch between the confocal and temporal gates, we first determined how much the entire reference arm must be shifted for each micrometre of sample movement. This was done by moving the sample 30 µm and adjusting the motorized translation stage in the reference arm to find the position that gave the highest image contrast. From this, we calculated the required reference arm shift per micrometre of sample displacement, assuming a homogeneous axial refractive index within the sample.

### Biological samples

For imaging, we used freshly excised organs of C57BL/6 mice provided by Urtė Neniškytė’s group (Life Sciences Centre, Vilnius University). Our laboratory did not perform any animal procedures. All animal work—including breeding, housing, and tissue collection—was performed at the Life Sciences Centre animal facility, in accordance with Directive 2010/63/EU, and approved by the Lithuanian State Food and Veterinary Service (permit No. G2-92). Only the excised organs were transferred to our laboratory for imaging; no additional animal procedures were conducted at Centre for Physical Sciences and Technology.

Tissue was kept in PBS and imaged by slightly pressing the tissue surface against the 100 µm-thick microscopy coverslip glass plate to which the objective was coupled using immersion oil.

## Supplementary information

dynamic full-field OCT Auksorius 2026 Suppl

Movie S1

Movie S2

Movie S3

Movie S4

Movie S5

Movie S6

Movie S7

Movie S8

## Data Availability

The datasets generated and/or analysed during the current study are not publicly available due to their large size but are available from the corresponding author on reasonable request.

## Code availability

Custom Labview-based code was used for data acquisition and processing in this study, but it is not publicly available.
