# Supplementary material for: High-resolution dynamic full-field optical coherence microscopy: illuminating intracellular activity in deep tissue
Source: Npj Imaging. 2026 Mar 31;4:21. doi: 10.1038/s44303-026-00153-y (PMC13039945; doi:10.1038/s44303-026-00153-y)
Supplement: Supplementary file 1 — dynamic full-field OCT Auksorius 2026 Suppl [file 44303_2026_153_MOESM1_ESM.pdf]

Resolution characterisation

The theoretical lateral resolution of the FF-OCM system is diffraction-limited to approximately Δx = 268 nm at the central wavelength, λ of 550 nm, calculated using the Rayleigh criterion ($\Delta x = 0.61\lambda/NA$) with NA=1.25. With a total magnification of 100× and camera pixel size of 12 µm, the effective pixel size in sample plane is 120 nm, supporting minimal Nyquist sampling at ~240 nm (strictly requiring at least 2 pixels per resolvable feature to avoid aliasing) and confirming the system is near the optical limit without significant aliasing. The smallest fully resolvable features in USAF target correspond to 274 nm bar spacing (element 6, group 10), as shown in Fig. S1a. Axial resolution was determined by measuring the full-width-half-maximum (FWHM) of an axial profile obtained from a mirror sample, shown in Fig. S1b.

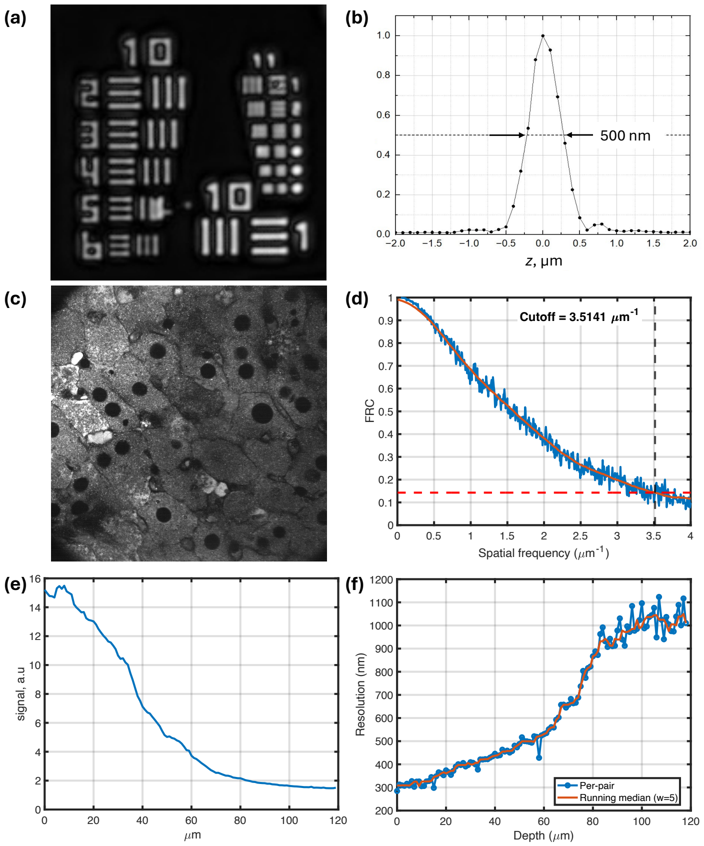

Fig. S1. Resolution characterization of the Full-Field Optical Coherence Microscopy (FF-OCM) system. (a) FF-OCM image of the USAF 1951 test target. (b) Axial resolution measurement. (c) Dynamic FF-OCM image of a live liver tissue derived with STD method and used for resolution determination with FRC. (d) FRC as a function of spatial frequency used to determine lateral resolution of 285 nm. (e) Dynamic FF-OCM signal as a function of imaging depth. (f) Resolution as a function of imaging depth as determined by the FRC method.

We have also estimated the resolution of *d*-FF-OCM image that was derived through STD analysis of a liver, shown in Fig. S1c, by using Fourier Ring Correlation (FRC). Fig. S1d shows FRC as a function of spatial frequency. It demonstrates resolution of ~285 nm, which is obtained when the FRC threshold is set to a standard value of 1/7 (0.143) resulting in the spatial frequency of 3.51 µm^{-1} (or ~285 nm). Fig. S1f shows resolution as a function of imaging depth.

Imaging depth demonstration

Imaging depth in liver tissue with the developed *d*-FF-OCM system can exceed 100 µm, as shown in the Fig. S2.

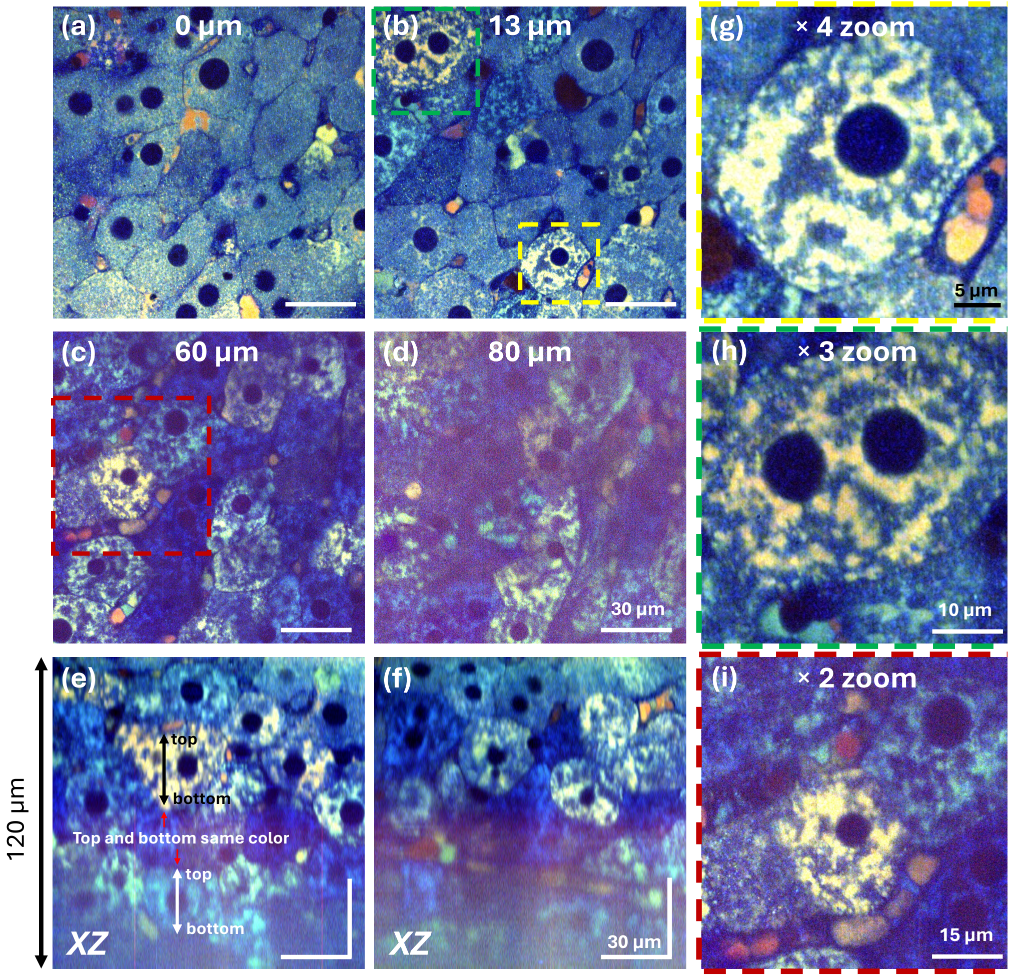

Fig. S2. Dynamic FF-OCM enables high-contrast en face imaging of freshly excised mouse liver down to 120 µm depth. Panels (a–d) show en face views at 0 µm, 13 µm, 60 µm, and 80 µm depths—note the preserved contrast even at 80 µm. Panels (e) and (f) present the full axial (*XZ*) span with 5–6 layers of hepatocytes that were derived by averaging 20 consecutive *XZ* images (B-scans). Panel (e) also indicates that the dynamic signal does not change between the top and the bottom of the cells. Panels (g–i) are 4× magnified detail views highlighting hepatocyte plates and blood-filled sinusoids. Dynamic vibration frequencies are color-coded as follows: blue (0.5–1 Hz), green (1.5–50 Hz), and red (50–150 Hz).

Visualizing Paneth Cells

Paneth cells and their granules are clearly visible in fixed mouse tissue (Fig. S3a-b), with greater clarity than in fresh *ex vivo* tissue (Fig. 4c). See Movie S6 for the full movie. Recorded with white LED (MCWHL7, Thorlabs).

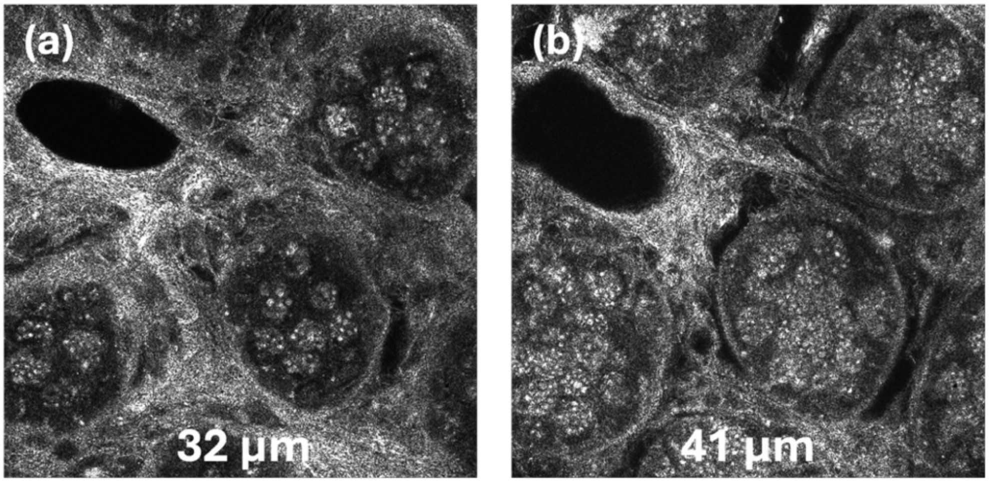

Fig. S3. Paneth cells featuring granules imaged with static FF-OCM on fixed mouse gut at different depth (a) 32 µm and (b) 41 µm.

Comparison between the new and conventional light sources

We compared the new light source used in this work (LS–WL1, Lightsource.tech, Germany) with a white LED (MCWHL7, Thorlabs). FF-OCM images were acquired without averaging. At an imaging depth of 55 µm, the LED images show little discernible structure, whereas images acquired at the same depth with the new source clearly retain structural detail.

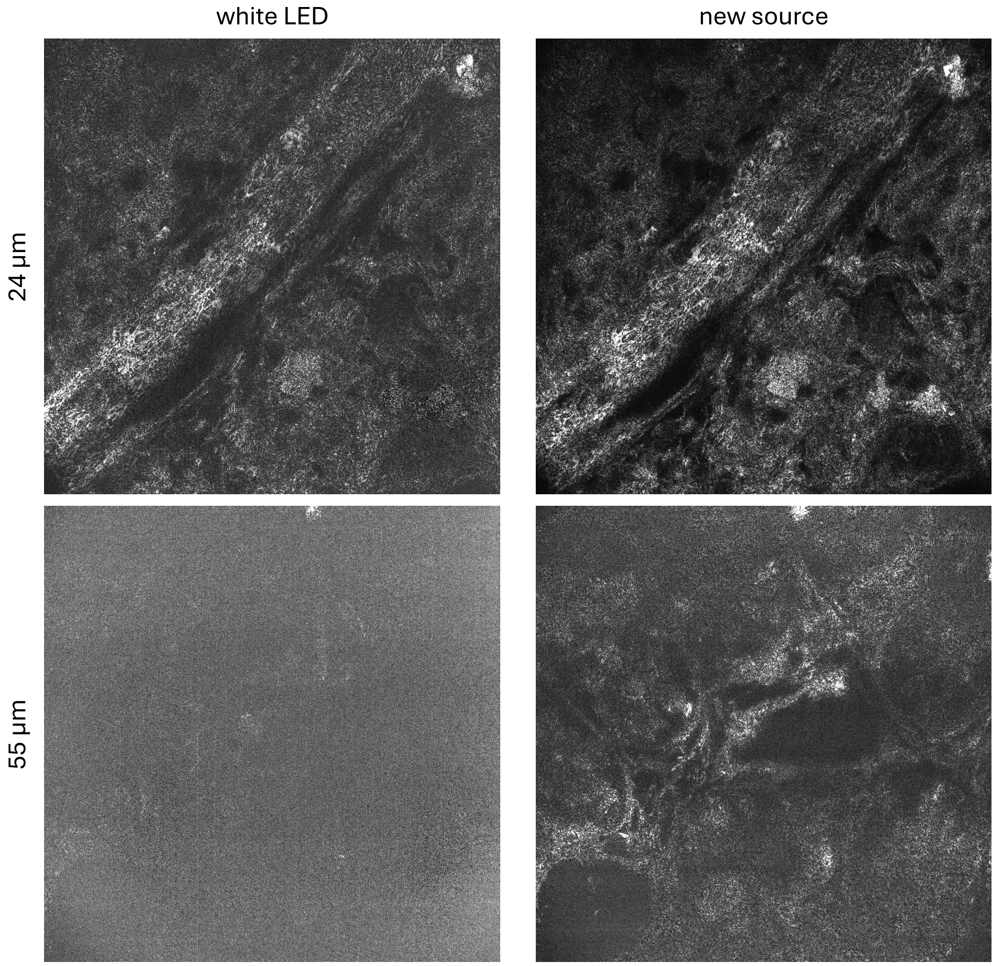

Fig. S4. Mouse intestine FF-OCM images acquired with the new (right column) and 'old' (left column) light sources at the depth of 24 µm (top row) and 55 µm (bottom row).

Frequency extension to 250 Hz

Extension of frequency analysis band from the upper cut-off frequency of 30 Hz to 250 Hz yields more contrasted images in mouse intestine and liver tissue, as shown in Fig. S5.

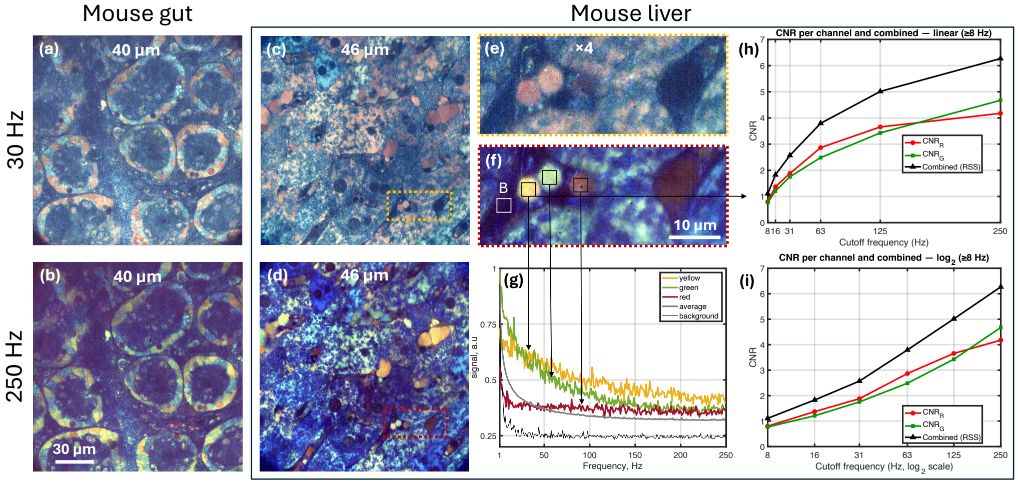

Fig. S5. Raising the upper frequency limit from 30 Hz to 250 Hz significantly improves contrast in *d*-FF-OCM images. Panels (a) and (b) show mouse gut images derived with 30 Hz and 250 Hz cut-off frequencies, respectively, and panels (c) and (d) show the corresponding liver images. Panels (e) and (f) are 4× magnified views of the liver regions highlighted by the yellow and red boxes in (c) and (d), respectively. Panel (g) plots the frequency spectra of three structures, 4-5 µm in diameter, within the liver sinusoids in (f). Average and background frequency spectra are also shown. Note that the dark red/brown structures visible in (f) are absent in the 30 Hz image (e) because its high-frequency components are filtered out by the 30 Hz analysis. (h) CNR as a function of cut-off frequency on the linear scale and on the log scale (i) derived from the yellow structure as a signal and white box (labelled B) as a background in (f).

We quantified visibility using the contrast-to-noise ratio (CNR) in the red and green channels with fixed signal and background ROIs (images analyzed in linear RGB). For each channel CNR was computed as: CNR = $(\mu_s - \mu_b)/\mathrm{sqrt}(\sigma^2_s + \sigma^2_b)$, where μ_s is mean signal, μ_b – mean background, σ_s – STD of signal, σ_b – STD of background We also reported a single combined value assuming negligible cross-channel noise correlation: $CNR_c = \mathrm{sqrt}(CNR^2_{red} + CNR^2_{green})$. After fixing the upper frequency at 250 Hz, we examined alternative partitions of the frequency ranges across channels. Figure S6a–c shows RGB images produced with different channel windows. Figure S6c also presents a mean-normalized RGB image with equalized channel intensities. To achieve this, we integrated 1–26 Hz (blue), 26–96 Hz (green), and 96–250 Hz (red), as summarized in Figure S6d.

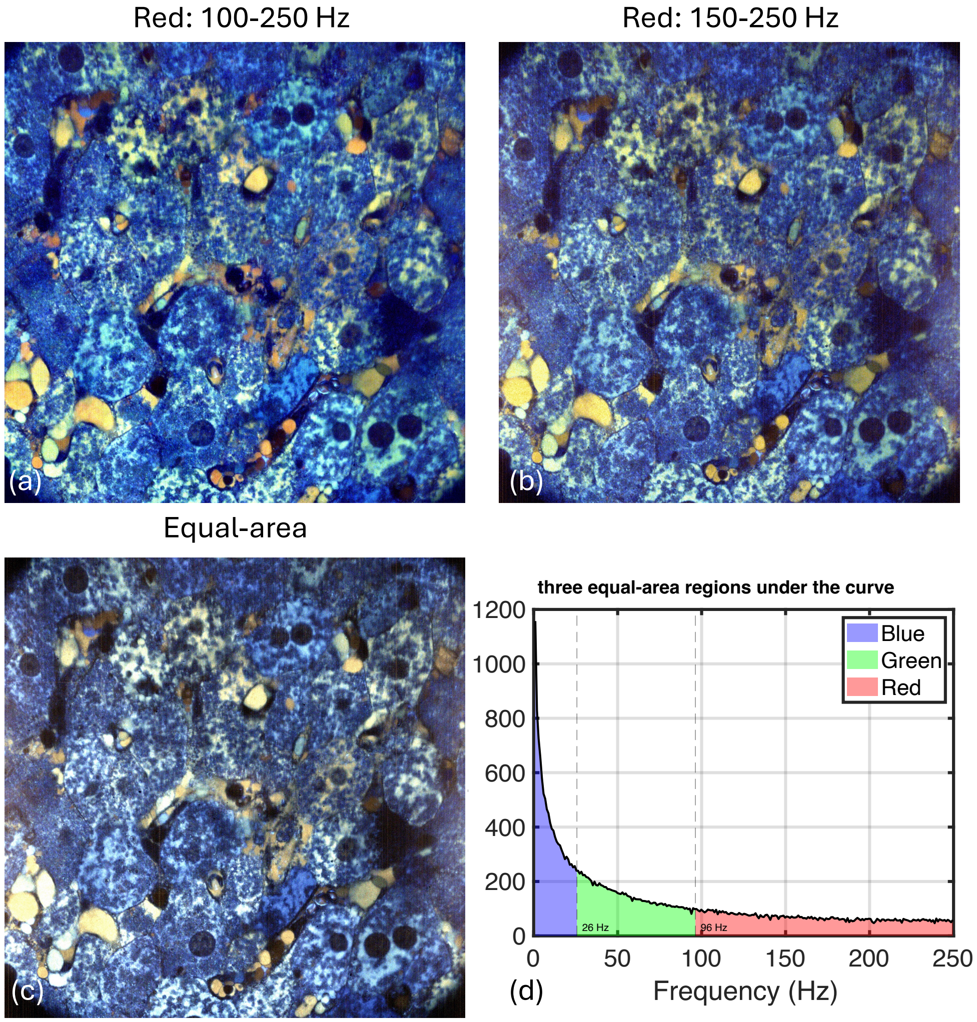

Fig. S6. Dynamic image comparison with different frequency ranges. (a) Blue: 1–3 Hz, Green: 3–100 Hz, Red: 100–250 Hz. (b) Blue: 1–3 Hz, Green: 3–150 Hz, Red: 150–250 Hz. (c) Blue: 1–26 Hz, Green: 26–96 Hz, Red: 96–250 Hz. (d) frequency spectrum of image in panel (c) and frequency ranges of RGB channels.

Supplementary Movies:

Movie S1: 3D volumetric rendering of ex vivo mouse liver acquired by dynamic FF-OCM, visualizing hepatic architecture and subcellular dynamics through a 100 µm depth stack.
Movie S2: Depth-resolved *en face* sequence of *ex vivo* mouse liver captured by dynamic FF-OCM, illustrating tissue architecture and subcellular dynamics across multiple z-planes.
Movie S3: Depth-resolved *en face* sequence of *ex vivo* murine small intestine (sample #1), captured from the mucosal side by dynamic FF-OCM, illustrating epithelial and lamina propria structures across multiple z-planes.
Movie S4: Depth-resolved *en face* sequence of *ex vivo* murine small intestine (sample #2), captured from the mucosal side by dynamic FF-OCM, illustrating epithelial and lamina propria structures across multiple z-planes.
Movie S5: Depth-resolved *en face* sequence of *ex vivo* murine small intestine, captured from the serosal side by dynamic FF-OCM, illustrating longitudinal and circular muscle, myenteric and submucosal plexuses and crypts.
Movie S6: Depth-resolved *en face* sequence of *ex vivo* murine small intestine, captured from the serosal side by static FF-OCM, illustrating longitudinal and circular muscle, myenteric plexus and Paneth cells with clear granule structure.
Movie S7: Dynamic RGB images of a liver generated as a function of cut-off frequency varying from 2 Hz to 250 Hz.
Movie S8: Dynamic RGB images of a liver generated as a function of threshold frequency between the green and red channels varying from 10 Hz to 240 Hz.
